# Supplementary material for: Ecological design of augmentation improves helicopter ship landing maneuvers: An approach in augmented virtuality
Source: PLoS One. 2021 Aug 11;16(8):e0255779. doi: 10.1371/journal.pone.0255779 (PMC8357182; doi:10.1371/journal.pone.0255779)
Supplement: S1 File — (DOCX) [file pone.0255779.s001.docx]

# Supplementary Information

Ecological design of augmentation improves helicopter ship landing maneuvers: an approach in augmented virtuality

## Control of perceptual learning during the Familiarization phase

Figure S1 depicts the changes in the total duration of the maneuver during the unfolding of the Familiarization and Experiment phases. A one-way RM-ANOVA performed on the individual average values of the total duration of the maneuver during the familiarization phase and during each experimental block of 6 trials revealed a significant main effect of the block repetition (F(6, 90)=3.9214, p<0.05, *η_p_*^2^= 0.21). The total duration of the maneuver was significantly longer during the Familiarization phase than during the 1^st^ experimental block (p<0.05). The total duration of the maneuver does not significantly differed between the 1^st^ and the 6^th^ experimental block (p>0.05). Therefore, the familiarization phase was long enough to allow participants calibrate themselves with the task and *Augmentations*.


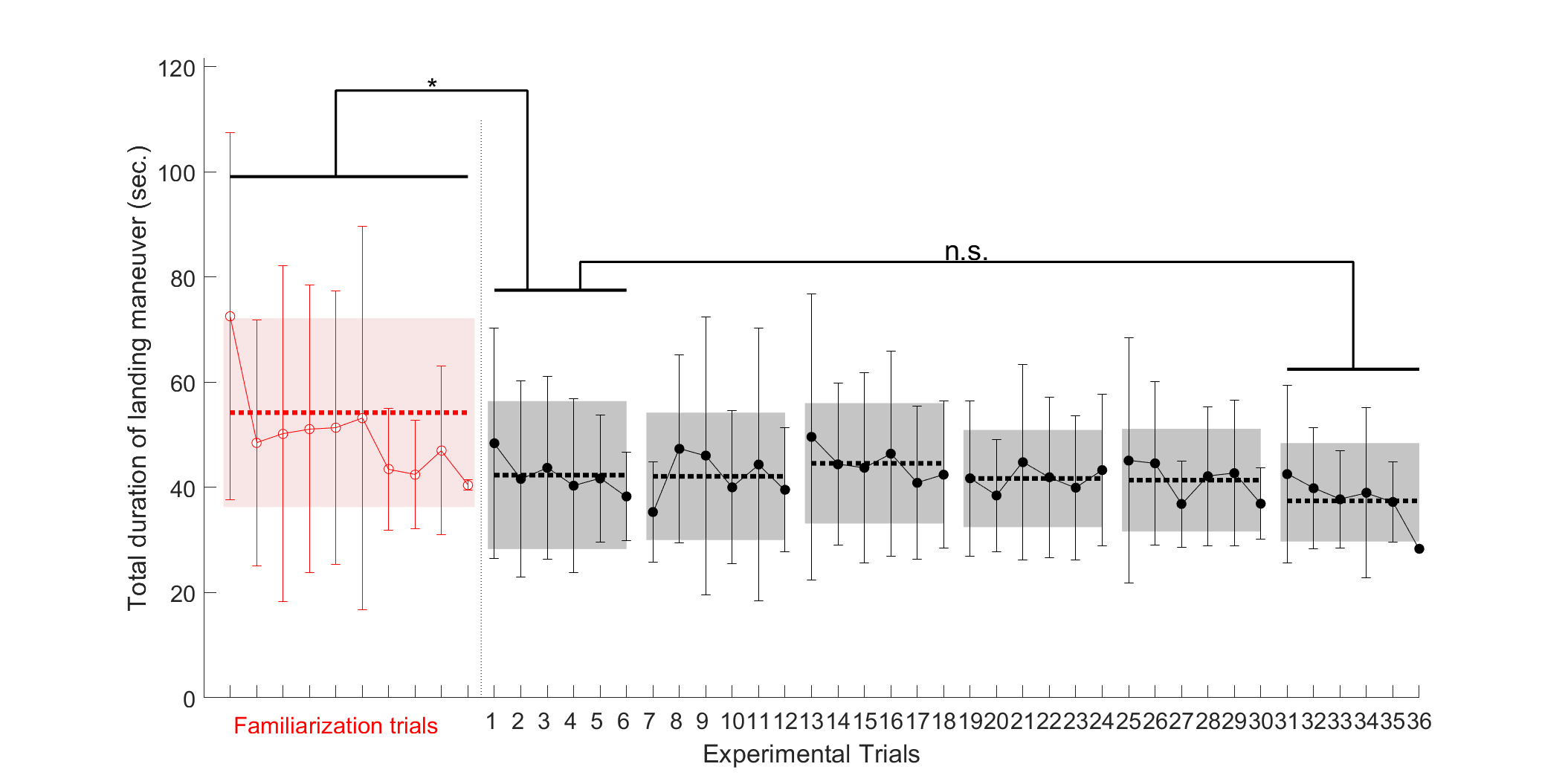


Fig. S1. Inter-individual average total duration of the landing maneuver expressed as a function of the unfolding of trials during the experiment. Trials performed during the Familiarization phase are depicted with empty circles while trials performed during the Experiment phase are depicted with full circles. The horizontal dotted lines depict the average total duration of the maneuver for each block and colored areas depict the standard deviation of inter-individual values. Vertical bars depict standard deviation of inter-individual average values of total duration for the considered trial.

## Workload Level


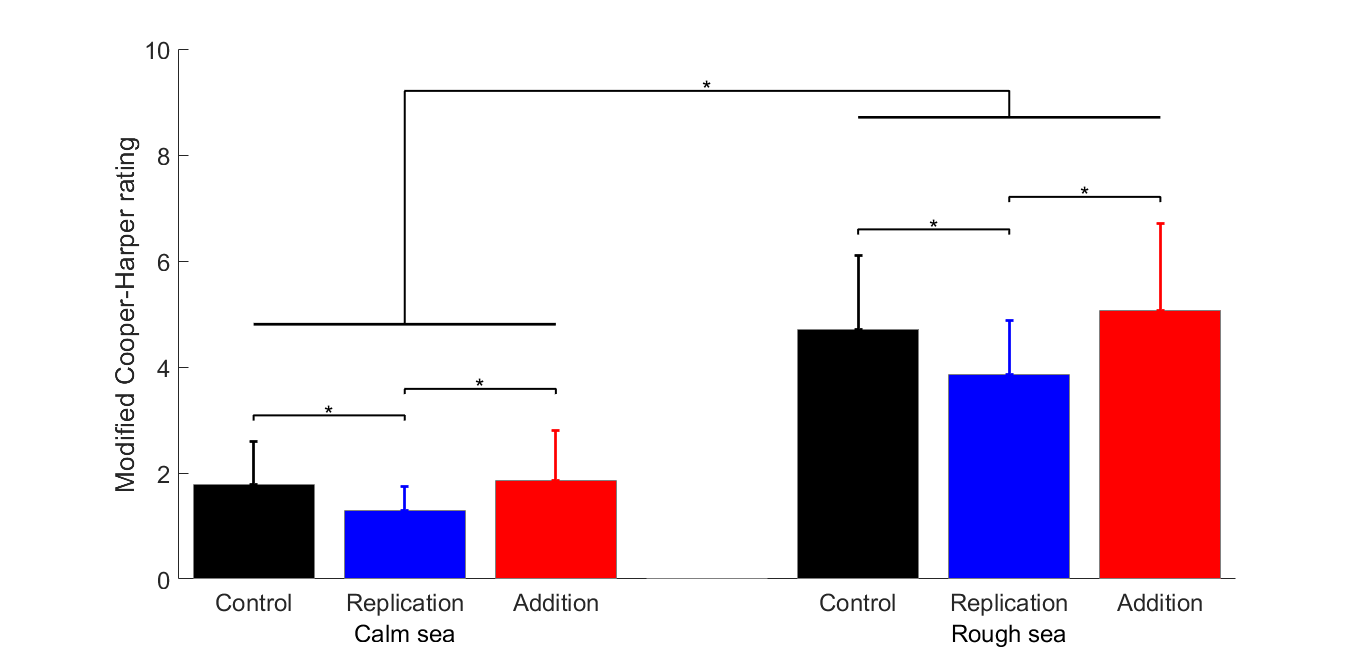


Fig. S2. Inter-individual average ratings gained with the Cooper-Harper rating scale in the two environments (Calm, Rough sea) and for the three augmentations (Control, Replication, Addition). Vertical bars depict standard deviation of inter-individual average values.

## Thrust Commands Level

### Backward displacements.

Figure S3A depicts the changes in the occurrence of backward displacements with manipulations of *Environment* and *Augmentations*. A 2-way RM-ANOVA performed on the individual average values of occurrence of backward displacements firstly revealed a significant main effect of the *Environment* factor (F(1,30)=11.63, p<0.05, *η_p_*^2^=0.28). The occurrence of the backward displacements significantly increased in *Rough* as compared to *Calm* sea (5.00*±*2.96 vs. 3.80*±*2.74, p< 0.05). No significant effect of the *Augmentation* factor was observed (F(2,30)=0.01, p>0.05, *η_p_*^2^=0) but the ANOVA revealed a significant *Environment*×*Augmentation* interaction (F(2,30)=4.32, p<0.05, *η_p_*^2^=0.22). Post-hoc tests revealed, in *Rough* sea, a higher occurrence of backward displacements in the *Control* than in the *Replication* and *Addition* conditions (5.86*±*3.47 vs. 4.57*±*2.62, p<0.05). In parallel, the same pattern of results was observed with the cumulative duration of backward displacements. To summarize, the *Replication* and *Addition* conditions permitted the reduction of the occurrence and duration of the backward displacements. These variables, however, do not mirror the greater benefits of the *Addition* in comparison to those of the *Replication* one.

### Actions on collective stick.

Given that the virtual rotorcraft was driven in velocity mode (quite similar to an actual helicopter without high-level control laws), a reversal movement of the joystick can be performed without producing a backward displacement. For instance, moving the collective stick from 100% to 75% of its travel will only cause the helicopter to decelerate. Therefore, the investigation of collective stick reversal movements offers a deeper insight concerning the potential benefits of augmentation conditions to the command of the helicopter. Figure S2B depicts the occurrence of collective stick reversal movements across environment and augmentation conditions. A 2-way RM-ANOVA performed on the individual average values of occurrences of collective reversal movements revealed a significant main effect of the *Environment* (F(1,30)=26.87, p<0.001, *η_p_*^2^=0.47). The number of occurrences of collective reversal movements increased from *Calm* to *Rough* seas (9.43*±*5.09 vs. 24.75*±*15.78, p<0.05). The ANOVA did not reveal any significant main effect of the Augmentation (F(2,30)=2.18, p>0.05, *η_p_*^2^=0.13) nor significant *Environment*×*Augmentation* interaction (F(2,30)=0.90, p>0.05, *η_p_*^2^=0.06) despite that the occurrence of collective stick reversal movements decreased in the *Replication* and *Addition* from about 20% and 22% as compared to the *Control* condition and from about 26% and 29% in *Calm* and *Rough* seas, respectively. A paired t-test was thus conducted to compare the individual average occurrence of collective stick reversal in *Rough* sea between the *Control* and *Addition* conditions. Results indicated a significant decrease of collective stick reversal movements in the *Addition* compared to the *Control* condition in *Rough* sea (22.18*±*15.84 vs. 28.74*±*16.67, t(15)=2.31, p<0.05). In summary, the *Addition* allowed a finer adjustment of the collective stick in comparison to the *Control* condition.


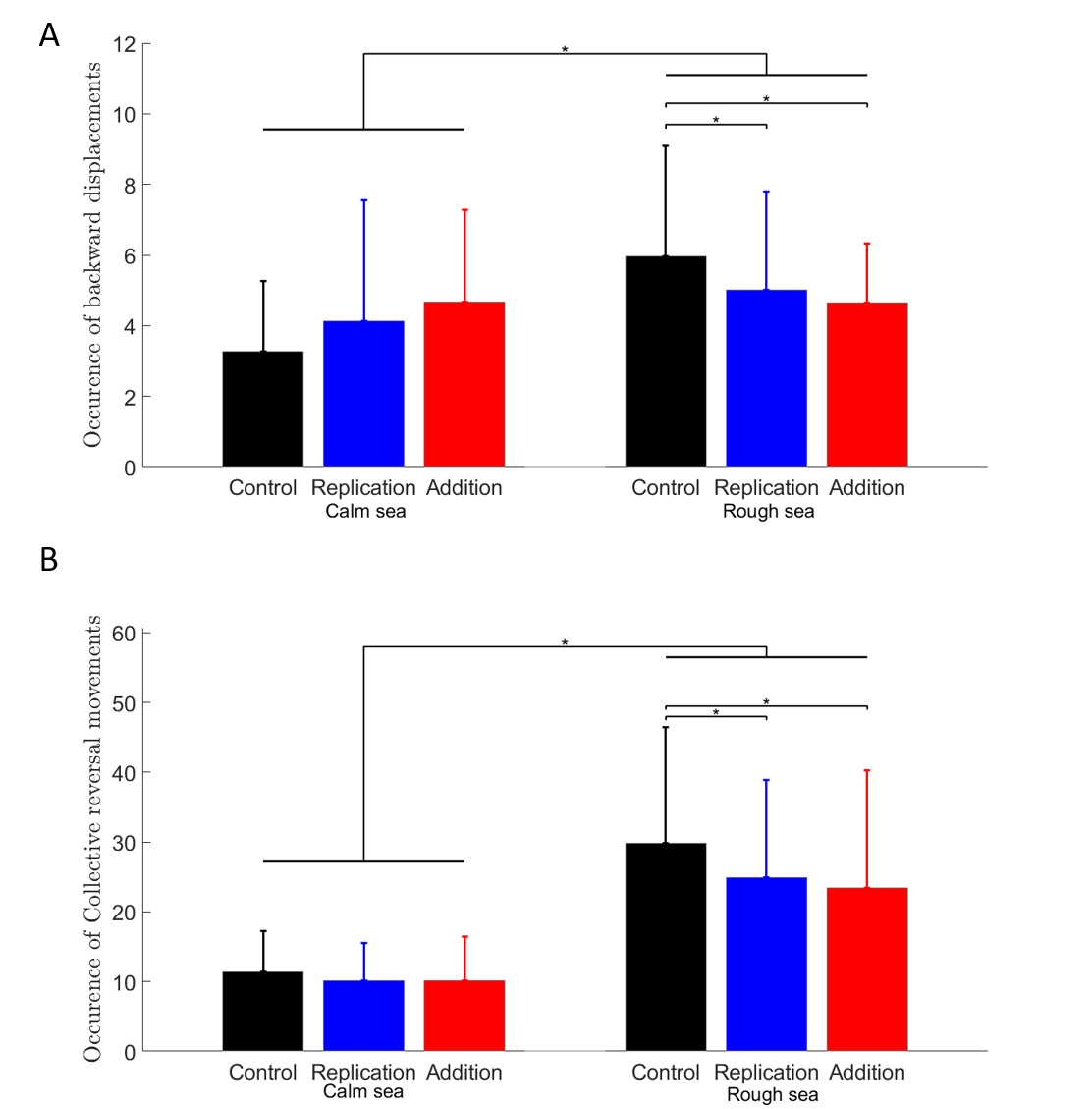


Fig. S3. Inter-individual average values of occurrence of backward displacements (A), and occurrence of collective stick reversal (B) during the landing phase. Vertical bars depict standard deviation of individual average values.
