# Supplementary material for: Ecological design of augmentation improves helicopter ship landing maneuvers: An approach in augmented virtuality
Source: PLoS One. 2021 Aug 11;16(8):e0255779. doi: 10.1371/journal.pone.0255779 (PMC8357182; doi:10.1371/journal.pone.0255779)
Supplement: Attachment — Submitted filename: PONE-D-20-29961_rev2_comments.docx [file pone.0255779.s003.docx]

Reviewer #1: Overall, it is well-written. I think it is unnecessary to include the abstraction hierarchy in the introduction if only one aspect is needed. There are different opinions. Either way would work for me.

Example:
Vicente, K. J., Moray, N., Lee, J. D., Hurecon, J. R., Jones, B. G., Brock, R., & Djemil, T. (1996). Evaluation of a Rankine cycle display for nuclear power plant monitoring and diagnosis. Human Factors, 38(3), 506-521.

***Thank you for sharing your feeling. We have summarized the results of the analysis provided by the abstraction hierarchy with a visual sketch. We hope this will help readers better understand the constraints of the work area.***

Reviewer #3: The authors extensively revised their manuscript, and, in doing so, addressed many of my concerns. I commend the authors for their efforts.

My remaining concerns are …

1) I think the readability of the manuscript would improve if the authors had a native English-speaker proofread/edit the manuscript.

***Thank you for your comment on the language. A professional, native English speaker have edited the reviewed manuscript and figures. We have proofread the manuscript again.***

2) The “What do display?” section discusses many topics, most of which do not have an obvious and direct connection to the displays the authors created/tested. I recommend the authors revise that section to make it more clear how what they described in that section led to the design of their displays.

***We removed content in this section with care since many topics were asked in previous comments. We clearly explained in the end of the paragraph how the abstraction hierarchy led to the design of our display. We moreover provided an addition sketch up summarizing the results of the analysis provided by the abstraction hierarchy.***

3) In the “How to display?” Section, it is still not as clear as it could be how the authors’ displays are “ecological”. That is particularly true for the “Replication” display. The section gives the impression that “ecological” mainly applies to the “Addition” display, and may not apply very much (or at all) to the “Replication” display. More generally, I could imagine both the “Replication” and “Addition” displays being designed by someone who does not utilize EID, and the content of this section does not convince me that framing these displays in terms of EID adds value.

***Indeed, we are convinced that addition augmentation is more ecological than replication augmentation (and the results attest to this) because not only it bypass the cockpit occlusion, but also it synthesizes tau-dot, an ecological invariant corresponding to the nature of deceleration relative to an approached surface. We reinforced this idea in the description of the “Replication” form.***

4) The paper’s title and Introduction focus heavily on EID. In contrast, the Discussion section surprisingly hardly mentions EID. That gives the impression that EID is not truly the focus, as suggested by the emphasis on EID in the title and Introduction.

## ***We framed more the existing*** ***section 4.2. Design of ecologically grounded Augmentations of the revised discussion on EID.***

5) The authors report that their displays affected certain aspects of landing behavior, and had no effect on other aspects of landing behavior. Despite that variability, they still state, in the Conclusions section (and perhaps elsewhere) that “… feeding the information-movement coupling with the tau variable on a gauge improved landing behavior …”. To me, that statement oversimplifies the situation. The “Addition” display improved certain aspects of landing behavior, but it had no effect on others. Further, some of the aspects of landing behavior that were unaffected presumably should have been affected. For example, the “Addition” displays seems well-suited to improve “Energy at impact”. However, the authors report that only the environment affected “Energy at impact”. When the authors make blanket statements like “The Addition displays improved landing behavior” they are glossing over such nuances. I think the paper would be improved if the authors adequately dealt with such nuances (those related to my examples, but also other reported analyses)

***The reviewer has fully understood the all the results reported. We followed the reviewer’s recommendations and removed the overstatements. As stated in the discussion of the revised manuscript, the Addition* « improved important aspects of landing behavior by significantly reducing the duration of landing maneuver and improving the load on rotorcraft commands. These improvements were probably favored by a finer perception of changes in current** $\dot{\boldsymbol{\tau}}$ **values that allowed in return finer actions on the collective stick». *These improvements are important to consider. Not only the first level of the abstraction hierarchy reveals that minimizing flight time allows to economize fuel (section 1.1.1 What to display ?, p. 7) but also improvements in rotorcraft command allows a decrease in the engine load that improves safety conditions for pilots by allowing them, with the thus freed engine capacity, a greater margin for action in case of dangerous situations (last § of section 4.2. Design of ecologically grounded Augmentations, p 39).***

***We also stated in the conclusion of the revised ms that significant improvements were not observed on* « aspects of landing behavior related to impact (i.e., relative phase of the touchdown, energy at impact) were not significantly improved by the Addition augmentation”. *The reviewer can admit that in the Rough Sea modality, where the minimization of impact energy is important to control because of the heave motion of the ship, the variables energy at impact and relative phase of the touchdown change, albeit very slightly, but still in the direction expected by the benefit brought by the augmentation. We attribute these results to the simplified helicopter model.***
